# A structural mapping of mutations causing succinyl-CoA:3-ketoacid CoA transferase (SCOT) deficiency

**DOI:** 10.1007/s10545-013-9589-z

**Published:** 2013-02-19

**Authors:** Naeem Shafqat, Kate L. Kavanagh, Jörn Oliver Sass, Ernst Christensen, Toshiyuki Fukao, Wen Hwa Lee, Udo Oppermann, Wyatt W. Yue

**Affiliations:** 1 Structural Genomics Consortium, University of Oxford, Oxford, OX3 7DQ UK; 2 Zentrum für Kinder- und Jugendmedizin, Universitätsklinikum Freiburg, 79106 Freiburg, Germany; 3 Klinische Chemie & Biochemie, Universitäts-Kinderspital, 8032 Zürich, Switzerland; 4 Department of Clinical Genetics, Juliane Marie Centre, 2100 Copenhagen, Denmark; 5 Department of Pediatrics, Graduate School of Medicine, Gifu University, Gifu, 501-1194 Japan; 6 Medical information Sciences Division, United Graduate School of Drug Discovery and Medical Information Sciences, Gifu University, Gifu, 501-1194 Japan; 7 Botnar Research Centre, Oxford Biomedical Research Unit, OX3 7LD Oxford, UK

## Abstract

Succinyl-CoA:3-ketoacid CoA transferase (SCOT) deficiency is a rare inherited metabolic disorder of ketone metabolism, characterized by ketoacidotic episodes and often permanent ketosis. To date there are ∼20 disease-associated alleles on the *OXCT1* gene that encodes the mitochondrial enzyme SCOT. SCOT catalyzes the first, rate-limiting step of ketone body utilization in peripheral tissues, by transferring a CoA moiety from succinyl-CoA to form acetoacetyl-CoA, for entry into the tricarboxylic acid cycle for energy production. We have determined the crystal structure of human SCOT, providing a molecular understanding of the reported mutations based on their potential structural effects. An interactive version of this manuscript (which may contain additional mutations appended after acceptance of this manuscript) may be found on the web address: http://www.thesgc.org/jimd/SCOT.

**Electronic supplementary material:**

The online version of this article (doi:10.1007/s10545-013-9589-z) contains supplementary material, which is available to authorized users.

## Introduction

Ketone bodies (acetoacetate, 3-hydroxybutyrate, acetone), predominantly produced in the liver, provide extrahepatic organs such as heart and brain with energy when glucose supply is limited (Sass 2012). Any enzyme malfunction in ketone body utilization (ketolysis) could lead to a buildup of unused ketone bodies and result in ketoacidosis. Succinyl-CoA:3-ketoacid CoA transferase (SCOT; gene name *OXCT1*; EC 2.8.3.5) catalyzes the first and rate-determining step of ketolysis (Fukao et al 2000; Kassovska-Bratinova et al 1996). SCOT is a member of the CoA transferase family I that activates acetoacetate by transferring a CoA moiety from succinyl-CoA to form acetoacetyl-CoA. Acetoacetyl-CoA is further metabolized by acetoacetyl-CoA thiolase into two acetyl-CoA molecules which enter the citric acid cycle for energy production (Williamson et al 1971). SCOT is a mitochondrial enzyme expressed in all extrahepatic tissues, but abundant in the heart, brain and kidney (Fukao et al 1997). There exists also a testis-specific isoform SCOT-t (gene name *OXCT2*), sharing 74 % amino acid identity with SCOT (Tanaka et al 2002).

Mutations in the human *OXCT1* gene on chromosome location 5p13 result in the rare autosomal recessive deficiency of SCOT (OMIM 245050) (Mitchell and Fukao 2001). SCOT-deficient patients, often with neonatal onset, present with recurrent ketoacidosis episodes that could be life-threatening, but with no symptoms between episodes (Niezen-Koning et al 1997). The number of reported cases is few, and symptoms, which may vary among individuals, include vomiting, lethargy and tachypnea, as well as unconsciousness caused by severe ketoacidosis (Berry et al 2001; Sakazaki et al 1995; Snyderman et al 1998). Permanent ketosis or ketonuria is a characteristic symptom but may be absent in patients retaining residual SCOT activity (Fukao et al 2010; Fukao et al 2011; Fukao et al 2004). Approximately 30 affected probands were reported to date and 24 mutations identified (Table 1). Here we report the crystal structure of human SCOT, and present an interactive mapping of missense mutations to understand the molecular basis of SCOT deficiency.

**Table 1:** Mutations reported for the human OXCT1 gene causing SCOT deficiency. Additional phenotype information can be found in the supplementary text

#	DNA change	Exon	Mutation site	Mutated residue	Protein change	Conservation	Reference
1^a,c,d^	c.112C > T^b^	2	Arg38	Cys	p.R38C		(Alkén 2008)
2^c^	c.335T > A^b^	4	Val112	Asp	p.V112D	Semi-Conserved	(Alkén 2008)
3	c.398T > A	4	Val133	Glu	p.V133E	Semi-Conserved	(Song et al 1998)
4	c.644C > T	6	Ala215	Val	p.A215V	Conserved	(Fukao et al 2011)
5	c.656G > A	6	Gly219	Glu	p.G219E	Conserved	(Fukao et al 2000)
6	c.661G > A	6	Val221	Met	p.V221M	Semi-Conserved	(Fukao et al 2000)
7	c.677G > A	7	Ser226	Asn	p.S226N	Semi-Conserved	(Fukao et al 2011)
8^c^	c.785C > G	8	Pro262	Arg	p.P262R	Conserved	Sass et al (unpublished)
9^c^	c.802C > T	8	Arg268	Cys	p.R268C	Conserved	Sass et al (unpublished)
10	c.803G > A	8	Arg268	His	p.R268H	Conserved	(Fukao et al 2007)
11	c.971G > A	10	Gly324	Glu	p.G324E	Conserved	(Fukao et al 2000)
12	c.980T > C	10	Leu327	Pro	p.L327P	Conserved	(Fukao et al 2011)
13^†^	c.1162A > G^b^	12	Met388	Val	p.M388V	Conserved	(Alkén 2008)
14	c.1210G > T	13	Val404	Phe	p.V404F	Conserved	(Fukao et al 2011)
15	c.1213T > C	13	Ser405	Pro	p.S405P	Conserved	(Fukao et al 2011)
16^†‡^	^b^	14	Leu429	Phe	p.L429F	Conserved	(Alkén 2008)
17	c.1304C > A	14	Thr435	Asn	p.T435N	Conserved	(Fukao et al 2010; Fukao et al 2004)
18	c.1367G > T	15	Cys456	Phe	p.C456F	Conserved	(Song et al 1998)
19	c.1402C > T	15	Arg468	Cys	p.R468C	Low-conserved	(Fukao et al 2011)
Insertion, deletion, frameshift mutations							
20	c.649C > T	6	Arg217	X	p.R217X	Semi-Conserved	(Longo et al 2004)
21	c.817G > T	8	Glu273	X	p.E273X	Variable	(Fukao et al 2011)
22	c.848C > G	9	Ser283	X	p.S283X	Conserved	(Yamada et al 2007)
23	c.658-666dup	6	Asn220-Ile222		p.N220-I222 dup	Conserved (Asn220), Semi-conserved (Val221,Ile222)	(Fukao et al 2010)
24	c.1561T > C^b^	6	X521R	Arg	Adding 20 AAs		(Alkén 2008)

^**a**^The R38C mutation resides in the N-terminus of the protein that is disordered and not modelled in the crystal structure

^b^DNA change in the R38C, V122D, M388V and X521R mutations are deduced from amino acid substitution. The DNA change in the L429F mutation is not reported and cannot be deduced from amino acid substitution

^c^These mutations have not been confirmed as pathogenic by expression analysis

^d^R38C and L429F mutations coexisted in one mutant allele

## Experimental procedures

### Expression, purification & crystallization

DNA fragment encoding the catalytic domain of human SCOT (aa 40–520; GenBank entry 4557817) was subcloned into pNIC-CTHF vector incorporating a C-terminal His_6_-tag. The plasmid was transformed into BL21(DE3)-R3-pRARE2, cultured in Terrific Broth at 37 °C, and induced with 0.5 mM IPTG. Cells were homogenized in lysis buffer (50 mM HEPES pH 7.5, 500 mM NaCl, 5 % glycerol, 5 mM imidazole, 1 mM PMSF, 0.5 mM TCEP), centrifuged to remove cell debris, and the supernatant was purified by Nickel affinity (HisTrap 1 ml GE/Amersham) and size exclusion (HiLoad 16/60 Superdex S200) chromatography. Purified protein was concentrated to 21 mg/ml and stored in 10 mM HEPES pH 7.5, 500 mM NaCl, 5 % (w/v) glycerol and 0.5 mM TCEP at −80 °C. Crystals were grown by vapour diffusion at 20 °C, in sitting drops mixing 100 nl protein pre-incubated with 2 mM acetyl-CoA and 300 nl reservoir solution containing 0.20 M sodium chloride, 0.1 M Tris pH 9.0 and 25 % (w/v) polyethylene glycol 3,350. Crystals were cryo-protected in mother liquor containing 20 % (w/v) glycerol and flash-frozen in liquid nitrogen.

### Data collection & structure determination

Diffraction data to maximum resolution of 2.20 Å were collected on beamline X10A at the Swiss Light Source, and processed using the CCP4 Program suite (CCP4 1994). SCOT crystallized in the P2_1_ space group with four molecules in the asymmetric unit (Supplementary Table 1). The structure of human SCOT was solved by molecular replacement with PHASER (McCoy et al 2005), using the pig heart structure as search model (PDB code 1M3E)(Bateman et al 2002). Initial automated model building was performed with ARP/wARP (Perrakis et al 2001), followed by cycles of iterative manual model building with COOT (Emsley and Cowtan 2004) and REFMAC5 refinement (Murshudov et al 1997). The refined model consists of protein residues 40–285 and 297–519. No electron density was observed for part of the inter-domain linker (residues 286–296). No ligand density for acetyl-CoA was found in the active site though it was added during crystallization. Structure factors and coordinates were deposited in the Protein Data Bank under the accession code 3DLX.

## Results and discussion

**Fig. 1:**
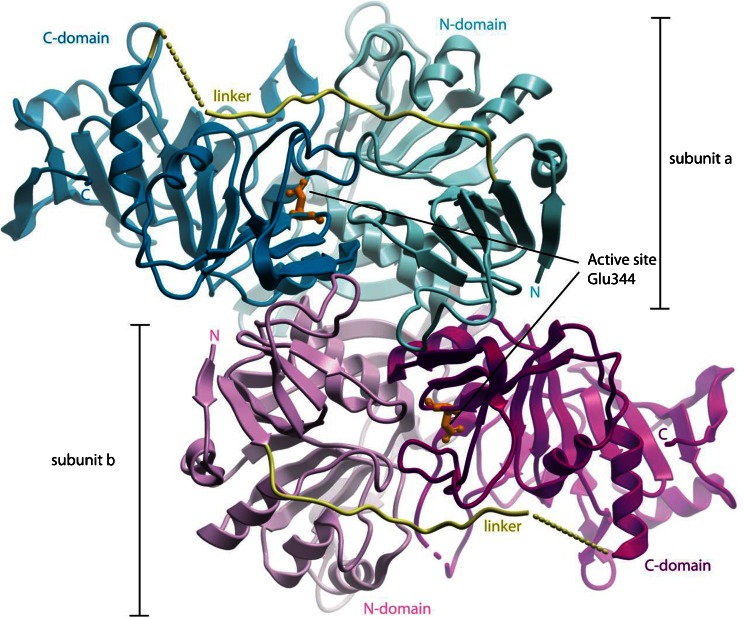
Ribbon diagram of human SCOT structure illustrating the homodimeric assembly. The two monomeric subunits **a** and **b** are coloured distinctively (*blue* and *magenta*). The active site in each subunit is indicated by the Glu344 residue shown in *orange sticks*

We have determined the crystal structure of human SCOT which exhibits a homodimer architecture containing two active sites (Fig. 1). Each monomer consists of the amino-terminal (N-; aa 40–272) and carboxy-terminal (C-; aa 298–510) domains, connected by a linker region (aa 273–297). The N- and C-domains share a common α/β structural fold for CoA transferase family I members (Heider 2001), as previously seen in the pig SCOT structure (89 % sequence identity)(Bateman et al 2002). The active site of each monomer is situated at the interface of the two domains, where a strictly conserved residue Glu344 (Fig. 1, orange sticks) attacks the incoming succinyl-CoA substrate and forms an enzyme-CoA thioester intermediate, as an integral part of the catalytic mechanism (Solomon and Jencks 1969). Residues in the active site of human SCOT are also conserved in the testis-specific isoform SCOT-t, suggesting it may have enzymatic activity.

**Fig. 2:**
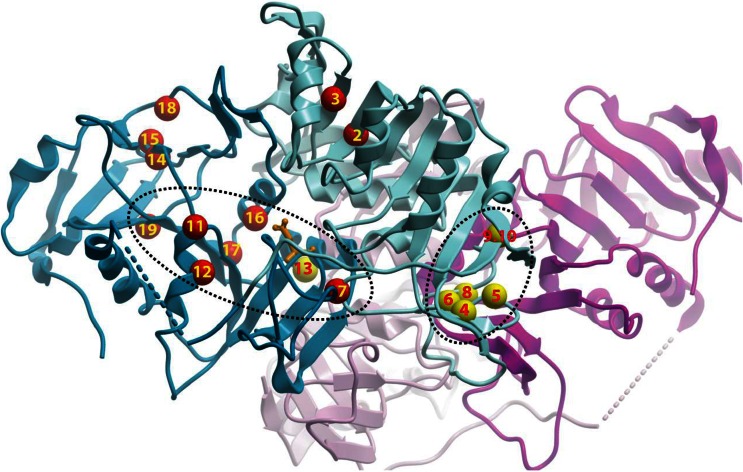
Clustering of human SCOT missense mutations, displayed in the same colour scheme as Fig. 1. The positions of amino acid mutations are indicated by *small spheres* and *numbered* according to Table 1. Mutations that affect the monomeric fold or dimerization interaction are coloured *red* and *yellow* respectively

To date, three nonsense, two insertion and 19 missense mutations leading to SCOT deficiency are known from literature or newly reported here (Table 1 and Supplementary text), although six missense mutations have not been confirmed as pathogenic mutations by expression analysis. There is a polymorphism c.173C > T (T58M) which retains full enzyme activity (Song et al 1998). The three nonsense mutations (R217X, E273X, S283X) are expected to cause premature translation termination, resulting in truncated SCOT proteins that lack completely the C-domain and hence abolish part of the active site. Another mutation c.1561T > C at the termination codon results in X521R and adds 20 amino acids in the C terminus of SCOT peptide (Alkén 2008). The missense mutations are broadly distributed between the N- and C-domains of the protein, although two clusters of ‘mutational hotspots’ can be observed (Fig. 2). One cluster is close to the interface between two SCOT subunits in a dimer. A duplication mutation (N220-I222dup) is also present in this region (Fukao et al 2010). The other cluster is located in secondary structure elements that make up the active site and CoA-binding site of the enzyme.

The mapping of missense mutations onto the human SCOT structure allows us to classify their potential structural consequences broadly into three types. In the first type, amino acids tightly packed in the enzyme are substituted to bulkier and/or charged residues (Fig. 2 and Table 1, *#2–7,11,14,16,17*). This likely results in severe steric and electrostatic clashes in the local environment which in turn can compromise severely the folding, architecture and stability of the enzyme. These mutants often result in the more severe phenotype (permanent acidosis), consistent with their much diminished enzyme activity (Fukao et al 2000; Song et al 1998). The second type disrupts the integrity of a secondary structure element, either by introducing a conformationally-restrained residue (e.g. Pro, Gly) into an α-helix/β-strand (Fig. 2 and Table 1, *#12,15*), or by removing such residues from their critical involvement in a loop/turn segment (Fig. 2 and Table 1, *#8*). The third type involves the substitution of arginine residues where their guanidinium side-chains are involved in salt bridge formation. These charged interactions contributed to stabilizing two neighbouring regions in 3D space that are distant apart in the polypeptide sequence. Substitution of arginine to a weakly positive-charged (Fig. 2 and Table 1, *#10*) or uncharged amino acid (Fig. 2 and Table 1, *#9,19*) will abolish these salt bridges. Though retaining partial enzyme activities, these mutant proteins are thermally less stable compared to wild-type (Fukao et al 2007; Fukao et al 2011).

## Electronic supplementary material

Below is the link to the electronic supplementary material.

ESM 1 (DOC 47 kb)

## Abbreviations

SCOT: Succinyl-CoA:3-ketoacid CoA transferase
OXCT1: 3-oxoacid CoA transferase 1
