# Supplementary material for: A structural mapping of mutations causing succinyl-CoA:3-ketoacid CoA transferase (SCOT) deficiency
Source: J Inherit Metab Dis. 2013 Feb 19;36(6):983–7. doi: 10.1007/s10545-013-9589-z (PMC3825524; doi:10.1007/s10545-013-9589-z)
Supplement: Supplementary file 1 — (DOC 47 kb) [file 10545_2013_9589_MOESM1_ESM.doc]

# A structural mapping of mutations causing Succinyl-CoA:3-ketoacid CoA transferase (SCOT) deficiency

Naeem Shafqat^1^, Kate L Kavanagh^1^, Jörn Oliver Sass^2,3^, Ernst Christensen^4^, Toshiyuki Fukao^5,6^, Wen Hwa Lee^1^, Udo Oppermann^1,7^ and Wyatt W. Yue^1*^

^1^ Structural Genomics Consortium, University of Oxford, UK OX3 7DQ

^2^ Zentrum für Kinder- und Jugendmedizin, Universitätsklinikum Freiburg, 79106, Freiburg, Germany

^3^ Klinische Chemie & Biochemie, Universitäts-Kinderspital, 8032 Zürich, Switzerland

^4^ Dept. of Clinical Genetics, Juliane Marie Centre, 2100 Copenhagen, Denmark

^5^ Department of Pediatrics, Graduate School of Medicine, Gifu University, Gifu 501-1194, Japan

^6^ Medical information Sciences Division, United Graduate School of Drug Discovery and Medical Information Sciences, Gifu University, Gifu 501-1194, Japan

^7^ Botnar Research Centre, Oxford Biomedical Research Unit, Oxford, UK OX3 7LD

**Supplementary Data**

**Supplementary Text** Phenotype information for Table 1

**Supplementary Table 1** X-ray data collection and refinement statistics

***Supplementary Text***

***Phenotype information for Table 1***

Most of the *OXCT1* mutations listed in Table 1 of main text and Supplementary Table 1 have been identified in patients with permanent ketosis. In some cases it is difficult to assess the impact of a single mutation *in vivo*, since it has only been identified in compound-heterozygosity: Mutations #1, #2, #6, mutations #8 and #9 and mutations #13 and #24 have only been identified in such a constellation.  Patients who were not characterized by a permanent ketosis, were homozygous for mutations V221M (#6 in Table 1), R268H (#10), L327P (#12) or T435N (#17).   A British girl homozygous for the V221M mutation came to medical attention at 22 months of age with otitis media and protracted vomiting, pallor, dehydration, and rapid, panting respirations (Fukao et al. 2000). At age 39 months, a second metabolic decompensation occurred. Later, under a regimen of avoiding prolonged fasting, dietary protein restriction (1.5 g/kg/day) and with oral sodium bicarbonate, if urinary ketones were positive, the patient experienced no further attacks. She showed normal growth and development at the age of 10 years. Two South African siblings homozygous for the  mutation R268H mutation  showed episodic ketoacidosis (new reference: Pretorius et al. 1996; Fukao et al. 2007). The R269H mutant protein was demonstrated to be temperature-sensitive (Fukao et al. 2007).  While one of the patients showed ketonuria on several occasions and was maintained on oral bicarbonate, the younger sibling remained asymptomatic following two decompensations and did not require such maintenance therapy. A 6 months old Croatian girl who was homozygous for the mutation T435N presented with appetite loss and coma, but did not show permanent ketonuria when she had recovered from the initial metabolic decompensation. Patients homozygous for the *OXCT1* mutation T435N have been reported not to show permanent ketosis, although their ketoacidotic crises were considered as severe as those of other SCOT deficient patients. T435N SCOT mutant enzyme is characterized by increased heat-sensitivity (Fukao et al. 2004).

**Supplementary Table 1: X-ray data collection and refinement statistics**

| **Data collection** | |
| --- | --- |
| Space group | P 2_1_ |
| *a*, *b*, *c* (Å) | 60.9, 168.5, 95.3 |
| *α*, *β*, *γ* (º) | 90.0, 105.9, 90.0 |
| Wavelength (Å) | 0.979 |
| Resolution (Å)^*^ | 48.1 (2.20) |
| R_merge_^*^ | 0.083 (0.526) |
| *I*/*σI*^*^ | 7.4 (2.0) |
| Completeness (%)^*^ | 98.6 (97.0) |
| Multiplicity^*^ | 2.3 (2.1) |
| **Refinement** | |
| Resolution (Å) | 48.1 (2.20) |
| No. reflections | 90754 |
| R_work_/R_free_ (%) | 17.9 (22.5) |
| *No. atoms* | |
| Protein | 13833 |
| Ligand/ion | 12 |
| Water | 375 |
| *B-factors (Å^2^)* | |
| Protein | 36.67 |
| Ligand/waters | 38.69/33.34 |
| *RMS deviations* | |
| Bond lengths (Å) | 0.016 |
| Bond angles (°) | 1.099 |
| PDB code | 3DLX |

^*^ Numbers in parentheses represent data in the highest resolution shell.
